# Cerebellar and Brainstem White Matter Geometric Alterations in Multiple System Atrophy: A DFA‐Based Biomarker for Disease Staging

**DOI:** 10.1111/cns.70623

**Published:** 2025-11-30

**Authors:** Hua Zhu, Shuxiang Zhu, Meixin Zhao, Ziyan Zhu, Yuxuan Shao, Xinxi Lu, Tao Liu, Haogang Zhu, Ni Shu, Hua Lin, Jian Cheng

**Affiliations:** ^1^ Beijing Advanced Innovation Center for Biomedical Engineering, School of Biological Science and Medical Engineering Beihang University Beijing China; ^2^ Department of Neurology, Xuanwu Hospital Capital Medical University Beijing China; ^3^ Department of Nuclear Medicine Peking University Third Hospital Beijing China; ^4^ School of Software Beihang University Beijing China; ^5^ School of Computer Science and Engineering Beihang University Beijing China; ^6^ State Key Laboratory of Cognitive Neuroscience and Learning & IDG/McGovern Institute for Brain Research Beijing Normal University Beijing Beijing China; ^7^ BABRI Centre Beijing Normal University Beijing China; ^8^ Beijing Key Laboratory of Brain Imaging and Connectomics Beijing Normal University Beijing China

**Keywords:** biomarker, brainstem, director field analysis, geometrics, multiple system atrophy, white matter

## Abstract

**Aims:**

To characterize white matter geometric pathology in cerebellar subtype of multiple system atrophy (MSA‐C) using director field analysis (DFA) and identify stage‐specific biomarkers.

**Methods:**

We analyzed single‐shell diffusion MRI (*b* = 1000) in 31 MSA‐C patients (15 early‐, 16 late‐stage) and 33 controls. DFA quantified axonal geometry (splay/bend/twist), complemented by fixel‐based analysis (FBA) and brainstem volumetry. Group comparisons used threshold free cluster enhancement (TFCE) (*p* < 0.05 FWE‐corrected). DFA‐altered regions were correlated with clinical scores. AutoGluon evaluated classification performance using different feature sets.

**Results:**

MSA‐C exhibited distinct geometric degeneration patterns: cerebellar pathways showed reduced splay, bend, and twist (reflecting Wallerian degeneration), whereas brainstem tracts demonstrated dissociated geometry (increased splay/bend but decreased twist). Brainstem twist reduction strongly differentiated early‐ and late‐stage MSA‐C (AUC = 0.95). Clinically, middle cerebellar peduncle bend correlated with motor progression (UMSARS‐II: *r* = 0.48), while cerebellar splay reduction predicted ataxia severity (SARA: *r* = −0.43).

**Conclusion:**

DFA captures circuit‐specific white matter pathology in MSA‐C, with brainstem twist emerging as a novel biomarker associated with disease stage. The integration of geometric metrics with automated machine learning provides a robust framework for early diagnosis and disease staging, highlighting distinct neurodegenerative mechanisms in cerebellar versus brainstem pathways.

## Graphical abstract

Director field analysis (DFA) uncovers spatially divergent white matter degeneration in MSA‐C: cerebellar fiber simplification (decreased splay/bend) reflects Wallerian degeneration, while brainstem twist reduction (decreased twist) tracks disease progression (AUC = 0.95). Multimodal DFA integration enables superior diagnosis (AUC = 0.98) and mechanistically targeted therapies.

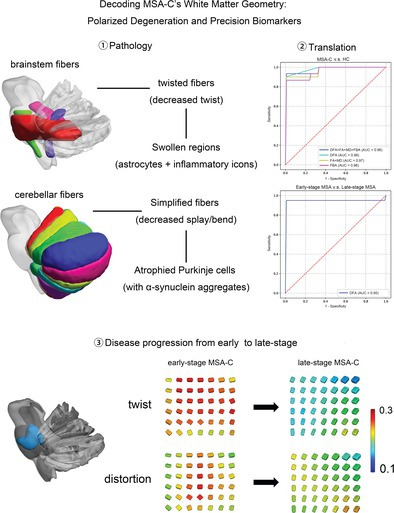

## 1 Introduction

Cerebellar subtype of multiple system atrophy (MSA‐C) is a progressive neurodegenerative disorder characterized by distinctive MRI findings including cerebellar atrophy and brainstem degeneration [1, 2, 3]. While current diagnostic criteria incorporate structural MRI findings (e.g., “hot cross bun” sign or putaminal atrophy) [4], their sensitivity remains limited in early disease stages [5]. Moreover, the lack of biomarkers capable of tracking pathological progression from initial autonomic failure [6] to end‐stage motor disability has severely hampered therapeutic development [7]. The development of biomarkers tracking microstructural changes from preclinical to advanced stages represents an unmet clinical need.

Director field analysis (DFA) [8] emerges as an ideal solution by directly mapping white matter geometric pathology—the very hallmark of α‐synuclein‐mediated axonal injury. Unlike traditional diffusion tensor imaging (DTI) or fixel‐based analysis (FBA) [9], which respectively quantify anisotropy or fiber density, DFA metrics (e.g., splay, bend, twist) provides inter‐voxel information which allows for quantifying the geometrics properties in a local spatial neighborhood. Pioneering study apply DFA metrics to AD groups have discovered unique geometric changes in brain white matter that are independent of diffusion tensor‐derived metrics [10], suggesting DFA's unique capacity to decode α‐synuclein's stereotypic attack on axonal topology in MSA‐C.

Prior investigations of MSA‐C white matter pathology face three fundamental limitations. First, reliance on FA/MD or FBA obscures distinct pathological processes—for example, increased fiber dispersion (elevated splay) cannot be distinguished from decreased fiber density using these methods. Second, most unimodal approaches [11, 12] fail to capture cross‐tissue pathological coupling—for example, they cannot reveal how white matter microstructural alterations interact with gray matter volume changes in driving clinical progression. Third, cross‐sectional designs preclude identification of progression‐specific signatures, as most studies pool early and late‐stage patients. Crucially, no study has systematically examined whether α‐synuclein‐induced axonal geometric changes correlate with clinical progression or multimodal imaging markers in MSA‐C.

In this cross‐sectional study, we applied DFA to map three‐dimensional geometric signatures in cerebellar‐brainstem pathways of 31 MSA‐C patients (stratified as 15 early and 16 late‐stage) and 33 age‐matched healthy controls, with a primary goal of identifying white matter geometric properties in MSA‐C. We hypothesize that region‐specific patterns of white matter geometric reorganization track pathological progression in MSA‐C, reflecting distinct underlying mechanisms of neurodegeneration. Based on the established vulnerability of cerebellar‐brainstem circuits in MSA‐C, we propose that cerebellar and brainstem fibers will exhibit distinct geometric signatures, corresponding to their differential patterns of neurodegeneration. Furthermore, we hypothesize that region‐specific geometric alterations track disease progression along connected neural pathways, potentially serving as sensitive staging biomarkers. By integrating DFA with FBA‐derived microstructural metrics and brainstem volumetry, we established a multimodal framework linking geometric pathology to clinical severity.

## 2 Materials and Methods

### 2.1 Participants

From July 2019 to March 2021, 33 healthy controls (HC) participants and 31 MSA‐C patients were recruited in this study. Among them, the MSA‐C patients were recruited from the Neurology Department of Xuanwu Hospital in Beijing, China (age 57.6 ± 7.3 years; 14 females). Age‐ and sex‐matched HC were enrolled from the community (age 57.5 ± 8.9 years; 12 females) Diagnoses were performed by experienced neurologists and inclusion criteria for patients were clinical diagnosis of possible or probable MSA‐C according to consensus criteria [5]. In all MSA‐C patients, early‐stage MSA‐C was defined as duration of disease ≤ 2 years based on ~25% of the median survival from the European (EMSA‐SG) [13], and American MSA‐C study groups (NAMSA‐SG) [14]. Longer duration (disease duration > 2 years) was divided into late‐stage MSA‐C. All late‐stage MSA‐C patients (*n* = 16) required walking assistance (walker or cane), with six being wheelchair‐bound. No early‐stage patients (*n* = 15) required mobility aids at the time of scanning, consistent with their clinical staging. This distribution aligns with expected disease progression patterns in MSA‐C.

The exclusion criteria were as follows: (1) current or previous history of other nervous system or systemic disease that might affect central nervous system integrity; (2) similar disorders in 1st‐ and 2nd‐degeree relative; (3) established acquired cause of ataxia; (4) evidence of a significant cognitive deficit (Mini‐Mental State Examination Score ≤ 24): (5) contraindications for MRI. The HC participants had a normal neurological examination without the history of neurological or psychiatric illness or head injury. Motor disabilities in MSA‐C patients were examined using the Motor Examination scores of the Unified MSA‐C Rating Scale, Part I (UMSARS‐I), Part II (UMSARS‐II), SARA and Disability Scale. The brief explanation of these clinical scale can be seen in Supporting Information 1 and 2. This study was approved by the Medical Research Ethics Committee of Xuanwu Hospital of Capital Medical University. All participants provided written informed consent before performing the study.

### 2.2 MRI Data Acquisition

All participants were imaged on a 3.0 T MRI scanner (Magnetom Skyra, Siemens, Germany). Three‐dimensional T1‐weighted images were obtained for each participant using magnetization‐prepared rapid gradient‐echo sequences with repetition time (TR) = 2530 ms, echo time (TE) = 2.98 ms, flip angle = 7°, voxel size = 1 × 1 × 1 mm^3^, field of view (FOV) = 256 × 256 mm^2^. Diffusion weighted imaging (DWI) data were acquired with the following parameters: TR = 11,800 ms, TE = 87 ms, flip angle = 90°, matrix = 128 × 128, voxel size = 1.8 × 1.8 × 2 mm^3^, gap = 0 mm, number of slices = 68. Diffusion sensitized signals were obtained by using 30 non‐colinear diffusion weighting directions with *b* = 1000s/mm^2^ and one image with *b* = 0.

### 2.3 MRI Data Processing

#### 2.3.1 Diffusion Data Pre‐Processing

Diffusion‐weighted MRI images were preprocessed using the FMRIB's Software Library (FSL, https://fsl.fmrib.ox.ac.uk/fsl) and MRtrix3 [15, 16]. Denoise was used to remove noise signals from raw DWI images. BET was then used to remove non‐brain tissue, and brain‐extracted images were visually inspected. Eddy correction was used to correct eddy current distortions and head movement. We adjusted the gradient directions based on the eddy current correction.

We used DMRITool (https://diffusionmritool.github.io) to reconstruct diffusion tensors and calculate FA, MD, and DFA metrics (splay, bend, twist, and total distortion). Detailed interpretations for DFA metrics have been described by Cheng and Basser [8], which are summarized in brief below.

#### 2.3.2 DFA Metrics

We used the novel mathematical framework DFA to compute the distortion of local white matter orientation based on tensor images. Based on previous studies [8], there are three types of geometric distortions, that is, splay, bend, and twist (Figure S1A). (i) Splay: spatial bending occurs perpendicular to the direction of the main molecular axis; (ii) bend: spatial bending is parallel to the direction of the main molecular axis; (iii) twist: neighboring directions are rotated with respect to one another, rather than aligned. (iv) The total distortion index (referred to as “distortion” in the following text) is a global measure of geometric distortion of WM tracts, and it is defined as the square root of the sum of squares of these metrics.

The three tensor fields in Figure S1A demonstrate splay, bend, and twist of the geometric distortions of white matter fiber in a local neighborhood. Figure S1B shows three synthetic tensor fields with their six index maps (splay, bend, twist, distortion, FA, and MD). All tensors in these three tensor fields have the same shape (e.g., FA or MD), but spatially different orientations. As such, using DFA metrics, we can detect the microstructural changes in the orientation of white matter fiber at an inter‐voxel level.

#### 2.3.3 Tract‐Based Spatial Statistics Analysis

Given the predominant cerebellar and brainstem pathology in MSA‐C, we restricted all analyses to these regions using anatomically constrained masks. The cerebellar regions of interest (ROIs) were defined by the Cerebellum atlas in FSL [17]. Brainstem ROIs were delineated using the brainstem tracts in JHU‐81 atlas [18]. The region contained in Cerebellum atlas and brainstem regions of the JHU‐81 white matter atlas can be seen in Figure S1C and Supporting Information 3. This region‐focused approach enhanced statistical power while mitigating multiple comparison penalties from whole‐brain analyses.

Mean FA maps were created and thinned to obtain a projection of all participants' FA data onto a mean FA skeleton that represented the centers of all tracts common to the group. Briefly, all participants' FA data were non‐linearly aligned to a standard template space (FMRIB58_FA) using FNIRT. Then, the mean FA image was created and thresholded (FA value > 0.2) to create the mean FA skeleton. Next, each participant's FA data was projected onto the thresholded mean FA skeleton. TBSS was also performed for the four DFA metrics (splay, bend, twist, and distortion) and MD. The displacement fields of the non‐linear registration estimated from the FA images were applied to warp DFA and MD maps. Then, the warped maps were subsequently projected onto the mean FA skeleton before applying voxel‐wise statistics.

#### 2.3.4 Fixel‐Based Analysis

In this study, we employed FBA to examine how geometric characteristics relate to white matter integrity in the brain microstructure of patients with MSA‐C. FBA is an advanced diffusion MRI framework that quantifies microstructural and connectivity properties of white matter at the level of individual fiber populations (fixels). For a detailed description of the FBA metrics and the analysis process, please refer to Supporting Information 4 and 5.

#### 2.3.5 Morphological Measurements

Considering the close pathological relationship between MSA‐C and brainstem regions, we divided the brainstem tissue into four subregions, calculated the brain tissue volume of these areas. We used an automated, non‐biased atlas‐based Bayesian segmentation procedure, applied in FreeSurfer v6.0 (http://surfer.nmr.mgh.harvard.edu), to derive quantitative estimates of brainstem and to label substructures tissue classes [19]. For a detailed description of the Freesurfer process, please refer to Supporting Information 6.

### 2.4 Statistical Analysis

#### 2.4.1 Voxel‐ and Fixel‐Wise Group Comparisons

To examine group differences in each diffusion metric (FA, MD, DFA, and FBA metrics) between HC and MSA‐C patients, as well as between early‐ and late‐stage MSA‐C subgroups, a general linear model was built with group factor as independent factors, demeaned age and gender as nuisance covariates. Voxel‐wise statistical analysis of white matter skeleton was conducted with permutation‐based nonparametric inference [20] on the FA, MD and DFA skeletonized maps using Randomize (5000 permutations) to assess group difference between MSA‐C and HC groups, and between early‐ and late‐stage MSA‐C subgroups. Threshold Free Cluster Enhancement (TFCE) [21] was used to correct for multiple comparisons across the whole cerebellum and brainstem (*p* < 0.05). Fixel‐wise statistical analysis was also performed using nonparametric permutation (5000 permutations) testing and connectivity‐based fixel enhancement (CFE) [22]. A family‐wise error corrected *p* value < 0.05 was considered statistically significant.

Given the relatively small sample size of early MSA‐C patients, a modified leave‐one‐out cross‐validation method was employed for comparison with HC group. Specifically, when conducting group comparisons between early‐stage MSA‐C group and HC group, one MSA patient from the early‐stage MSA‐C group was excluded, and the remaining early‐stage MSA‐C patients were compared with HC group. This process was repeated to ensure each patient was excluded once. In each comparison, voxel‐wise statistical analysis of white matter skeleton was conducted with permutation‐based nonparametric inference on the FA, MD and DFA metrics using Randomize to assess group difference between early‐stage MSA‐C group and HC group. TFCE was used to correct for multiple comparisons across the whole brain (*p* < 0.05). Finally, the white matter regions were identified and reported that consistently showed significant group differences across all 17 times comparisons.

Moreover, we performed between‐group statistics to compare the volume of three brainstem subregions (medulla, midbrain and pons) between the HC and MSA‐C groups. All continuous variables were assessed for normality using the Shapiro–Wilk test (*α* = 0.05). Data that met both normality (*p* ≥ 0.05) and homogeneity of variance assumptions (Levene's test *p* > 0.05) were analyzed using independent samples *t*‐tests. For data violating either assumption, the non‐parametric Mann–Whitney U test was employed instead. False discovery rate (FDR) correction was performed for multiple comparisons.

#### 2.4.2 White Matter Feature Extraction

Significant clusters from group comparisons (HC vs. MSA‐C; early‐ vs. late‐stage MSA‐C) were parcellated using FSL's JHU‐81 white matter atlas (cerebellum/brainstem regions). For atlas‐defined regions overlapping with significant clusters (*p* < 0.05 FWE‐corrected), mean diffusion metric values were extracted with specific inclusion criteria: regions > 100 contiguous voxels for FA/MD/FBA, and > 50 voxels for DFA. For DFA–FBA relationships, significant DFA (> 50 voxels) and FBA (> 100 voxels) maps were intersected to identify co‐altered regions. Retained atlas parcels required ≥ 30 voxels within the intersection. Region‐specific mean values (FA, MD, DFA, FBA) were extracted for behavioral correlations and machine learning classification. No missing data were present in the extracted feature sets for any subject; hence, no imputation was required.

#### 2.4.3 Correlation Analysis

To elucidate the clinico‐anatomical relevance of cerebellum and brainstem WM alterations in MSA‐C, we performed correlation analyses between DFA metrics and phenotypic measures using a tiered approach. For all white matter regions demonstrating significant between‐group differences (HC vs. MSA‐C, *p* < 0.05 TFCE‐corrected), we computed correlation between: (i) DFA metrics and clinical severity scores, (ii) DFA metrics and brainstem subregion volumes derived from T1 segmentation. All continuous variables were assessed for normality using the Shapiro–Wilk test (*α* = 0.05). Data that met both normality (*p* ≥ 0.05) and homogeneity of variance assumptions (Levene's test *p* > 0.05) were analyzed using Pearson's correlation. For data violating either assumption, the Spearman's correlation was employed instead.

Critically, regions exhibiting concurrent abnormalities in both DFA and FBA metrics underwent targeted co‐variation analysis. We first identified voxels surviving significance thresholds (DFA: > 50 voxels; FBA: > 100 voxels) in the HC versus MSA‐C contrast, then extracted mean values exclusively from their spatial intersection. These dual‐abnormality tracts were subjected to Pearson's correlation between DFA and FBA metrics. For all correlation analyses (clinical‐anatomical correlations and DFA‐FBA co‐variation), false discovery rate (FDR) correction was applied to adjust for multiple comparisons across tested regions/tracts, using the Benjamini–Hochberg procedure with a significance threshold of *p* < 0.05.

#### 2.4.4 Classification: Healthy Controls Versus Patients and Disease Progression Stages

To systematically assess the diagnostic and staging potential of DFA metrics, we introduced AutoGluon (version 0.5.2) [23] for automated machine learning within a rigorous two‐tiered data partitioning framework: First, an outer stratified 10‐fold cross‐validation divided the entire cohort (33 HC + 31 MSA‐C) into 10 folds while preserving group ratios, with each iteration reserving one fold as the completely independent test set and the remaining nine folds as the temporary training set. Second, within each temporary training set, AutoGluon implemented internal 10‐fold stratified bagging (auto_stack = True, num_bag_folds = 10) to train base models on 90% of the temporary data and generate out‐of‐fold (OOF) predictions on the held‐out 10%—these OOF predictions were used exclusively for constructing multi‐layer stacked ensembles, ensuring no data leakage to the independent test set. Feature sets were constructed from white matter regions exhibiting significant between‐group differences (Section 2.3), including four configurations: (i) FA/MD metrics; (ii) DFA metrics; (iii) FBA metrics; and (iv) their multimodal combination. AutoGluon was configured for maximal robustness: time_limits = 7200 (2‐h training budget per model), eval_metric = “roc_auc” (AUC optimization), and presets = “best_quality” (accuracy‐focused preset disabling hyperparameter tuning) (see Supporting Information 7). Post‐training, permutation feature importance was computed on each independent test fold. The average TPR and FPR across the 10 folds were then computed. The receiver operating characteristic (ROC) curve was plotted using the average TPR and FPR values, and the area under the curve (AUC) score was calculated for model verification and evaluation. The top 10 most important features for each classification were computed and listed. All AutoGluon analyses were conducted on a high‐performance computing server equipped with Intel i5‐9400F CPUs and 32 GB RAM. The 2‐h time limit per model was enforced using CPU‐based parallel processing.

## 3 Results

### 3.1 Clinical and Demographic Characteristics of the Patients

The MSA‐C patients and HC group had no significant differences in age and sex (both *p* > 0.05). Demographic and clinical characteristics of the MSA‐C patients are summarized in Table 1. Seventeen patients of the 31 were males and 14 were females. Mean age at symptom onset was 55.0 (SD 6.4) years; mean duration of symptoms was 2.7 (SD 1.7) years. According to consensus criteria, 19 patients fulfilled criteria for probable MSA (autonomic failure) and 12 patients for possible MSA (autonomic dysfunction). All patients with MSA‐C had cerebellar symptoms, and 19 had orthostatic hypotension. Twelve patients presented orthostatic discomfort such as dizziness resulting from hypoperfusion, and syncope likely occurred in two patients. Urinary symptoms were more common including incomplete bladder emptying (25 patients) and urinary incontinence (6 patients). Erectile dysfunction affected virtually most male patients, which is often the earliest symptom of MSA.

**TABLE 1:** Demographic characteristics and clinical measures of HC and MSA‐C patients.

Category	Characteristic	MSA‐C	HC
Demography	Number	31	33
	Age (years)	57.6 (7.3)	56.9 (5.9)
	Early‐stage	54.4 (7.6)	
	Late‐stage	60.3 (5.0)	
	Sex	17 males, 14 females	17 males, 16 females
	Disease duration (years)	2.7 (1.7)	
	Early‐stage	1.34 (0.57)	
	Late‐stage	4.07 (1.33)	
	Age of onset (years)	55.0 (6.4)	
Clinical measures	UMSARS‐I	13.7 (7.5)	
	UMSARS‐II	10.9 (6.0)	
	SARA	13.1 (7.7)	
	Disability Scale	1.81 (1.0)	

To identify the white matter microstructural characteristics across different stages of MSA, patients were divided into two subgroups: early‐stage MSA‐C (disease duration ≤ 2 years) and late‐stage MSA‐C (disease duration > 2 years). Among all the 31 MSA‐C patients, 15 were in early‐stage (duration range = 0.5–2 years) with the other 16 in late‐stage (duration range = 3–8 years). The average age of the early‐stage and late‐stage subgroups was 54 (range = 41–61) and 60 (range = 46–69) years, respectively, with the early‐stage patients being younger than those of the late‐stage group (*p* < 0.05). Among the 15 early‐stage patients, 8 were diagnosed for probable MSA‐C and 7 for possible MSA‐C. Among the 16 late‐stage patients, 11 were diagnosed for probable MSA‐C and 5 for possible MSA‐C.

### 3.2 Tract‐Based Spatial Statistics

**FIGURE 1:**
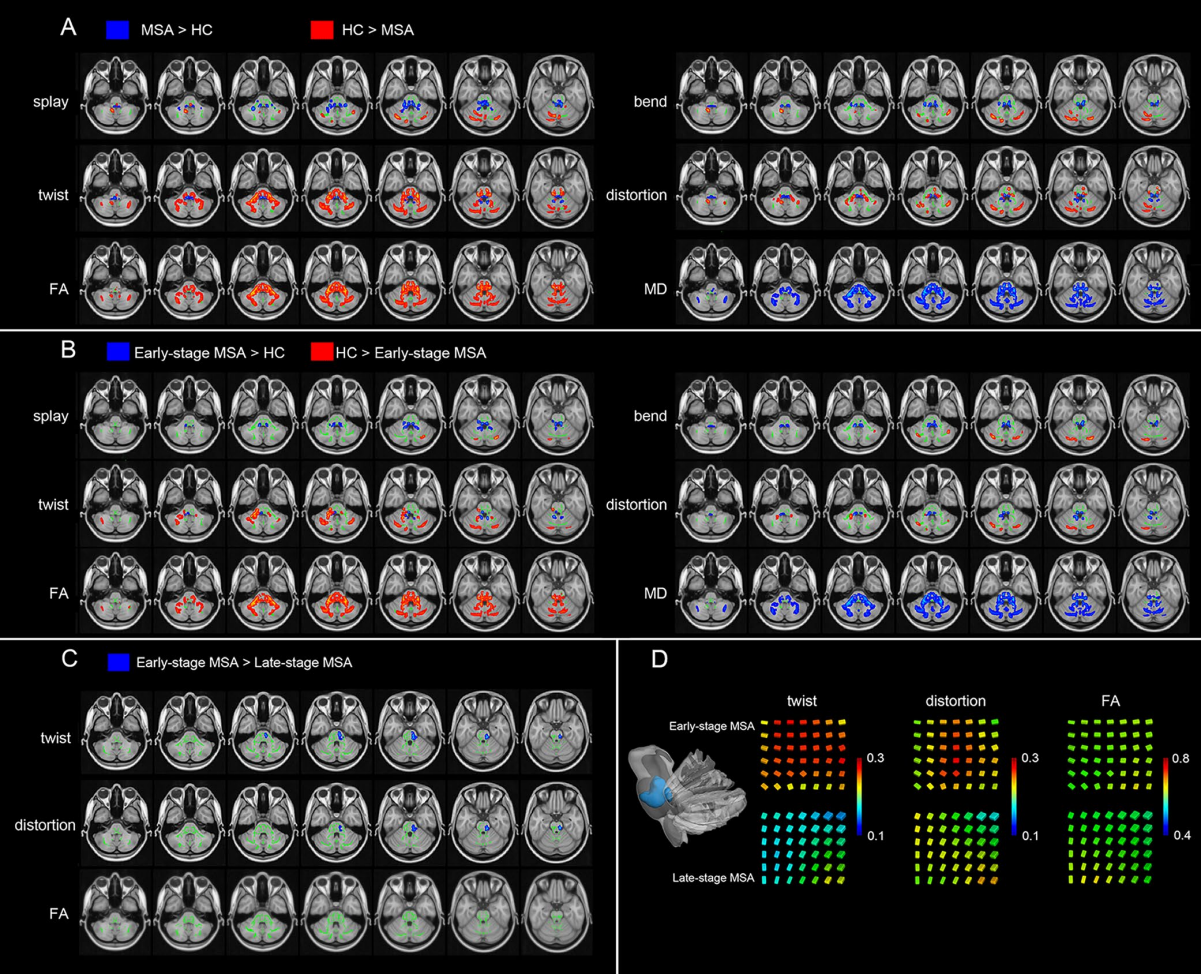
Tract‐based spatial statistics reveals staging‐specific white matter disruption in MSA‐C. (A) Tract‐based Spatial Statistics analysis showing a pattern of orientational changes (i.e., splay, bend, twist, and distortion) and classical metrics (i.e., FA and MD) overlaid on the white matter skeleton (green) in MSA‐C patients compared to HC group (*p* < 0.05, TFCE corrected). Blue is for the increase and red is for the decrease. (B) Tract‐based Spatial Statistics analysis showing a pattern of orientational changes (i.e., splay, bend, twist, and distortion) and classical metrics (i.e., FA and MD) overlaid on the white matter skeleton (green) in early‐stage MSA‐C patients compared to HC group (*p* < 0.05, TFCE corrected). Blue is for the increase and red is for the decrease. (C) Tract‐based spatial statistics analysis showing decreased twist and distortion overlaid on the white matter skeleton (green) in late‐stage MSA‐C patients compared to early‐stage MSA‐C patients (*p* < 0.05, TFCE corrected). Green denotes the white matter skeleton. Blue is for the decrease. (D) Sketch map of the significant decrease of the twist and distortion in the tensor field in late‐stage MSA‐C patients compared to early‐stage MSA‐C patients. The light blue cluster showed late‐stage MSA‐C patients had significantly different with early‐stage MSA‐C patients in twist and distortion but not in FA. We visualized the tensor field by coloring the glyphs using the twist, distortion and FA in one part of the significant different regions in two MSA‐C groups. We can visually observe the significant decrease of the twist and distortion in the tensor field in the given region where the FA is visually similar in two MSA‐C groups. Green denoted the white matter skeleton. FA, fractional anisotropy; HC, healthy controls; MD, mean diffusivity; MSA‐C, multiple system atrophy.

MSA‐C patients exhibited divergent patterns of white matter pathology in cerebellar and brainstem in Figure 1A. Within the cerebellum, widespread reductions in splay, bend, twist, and distortion metrics were observed: splay decreased in bilateral crus I and right IX; bend diminished in right IX, bilateral crus I, left V, and right VI; while twist and distortion showed parallel reductions in bilateral crus II, crus I, right IX, and VI (bilateral for twist; right for distortion). In contrast, brainstem pathology was predominantly characterized by increased geometric characteristics. Splay was elevated in the right inferior cerebellar peduncle (ICP), middle cerebellar peduncle (MCP), bilateral medial lemnisci (ML), and superior cerebellar peduncles (SCP), with concomitant increases in bend, twist, and distortion across ICP (bilateral), MCP, ML (bilateral), and SCP (left for twist; bilateral for distortion). Notably, opposing trends emerged in specific brainstem tracts: twist decreased in bilateral corticospinal tracts (CST), MCP, and pontine crossing tract (PCT), while distortion declined in MCP, PCT, and left CST, underscoring region‐dependent heterogeneity in white matter degeneration.

Distinct from the geometric pathology, conventional diffusion metrics revealed a spatially overlapping yet mechanistically divergent profile in MSA‐C patients compared to healthy controls (HC). FA was significantly reduced across cerebellar subregions (bilateral crus II, crus I, I–IV, IX, V, VI; vermis VI) and brainstem tracts (bilateral ML, CST, ICP, SCP, MCP, PCT). Conversely, MD exhibited marked increases in nearly identical regions, extending additionally to vermis VIIIa and right SCP.

Early‐stage MSA‐C patients exhibited divergent patterns of white matter pathology in cerebellar and brainstem in Figure 1B. Similarly, wide decrease in DFA metrics were observed in cerebellum: splay decreased in bilateral crus I; bend diminished in right crus II, bilateral crus I, and left V; while twist and distortion showed same reductions in right crus II, bilateral crus I, and right IX. In contrast, brainstem pathology was predominantly characterized by increased geometric characteristics. Splay and bend were elevated in the bilateral ICP, MCP, bilateral ML, PCT and SCP, with concomitant increases in twist, and distortion across ICP (right), ML (right for twist; bilateral for distortion), PCT (for distortion) and SCP (right for twist; bilateral for distortion).

Cross‐sectional comparisons between early‐ and late‐stage MSA‐C patients revealed spatially restricted differences in white matter geometrics (Figure 1C). Late‐stage MSA‐C patients exhibited significantly reduced twist and distortion metrics compared to early‐stage MSA‐C, localized to the MCP, PCT, and right CST. This selective reduction in fiber complexity—confined to brainstem pathways critical for sensorimotor and cerebellar connectivity—suggests that progressive simplification of fiber orientation in these regions may mark advanced disease states. Mechanistically, attenuated twist and distortion in MCP, PCT, and CST could reflect accelerated axonal degeneration with reduced branching complexity, potentially driven by α‐synuclein aggregation or microglial‐mediated pruning, though causal inferences require longitudinal validation.

As shown in Figure 1D, the light‐blue cluster showed where late‐stage MSA‐C patients had significantly lower twist and distortion metrics but not FA in comparison with early‐stage MSA‐C patients. We visualized the tensor fields by coloring the glyphs using the twist, distortion and FA in a part of the significant different regions in early‐ and late‐stage MSA‐C patients. We can visually observe distinct geometric microstructural changes in the given region, where twist and distortion metrics had a significant decrease while FA is visually similar in early‐ and late‐stage.

### 3.3 Fixel‐Based Microstructural Degeneration in MSA‐C

**FIGURE 2:**
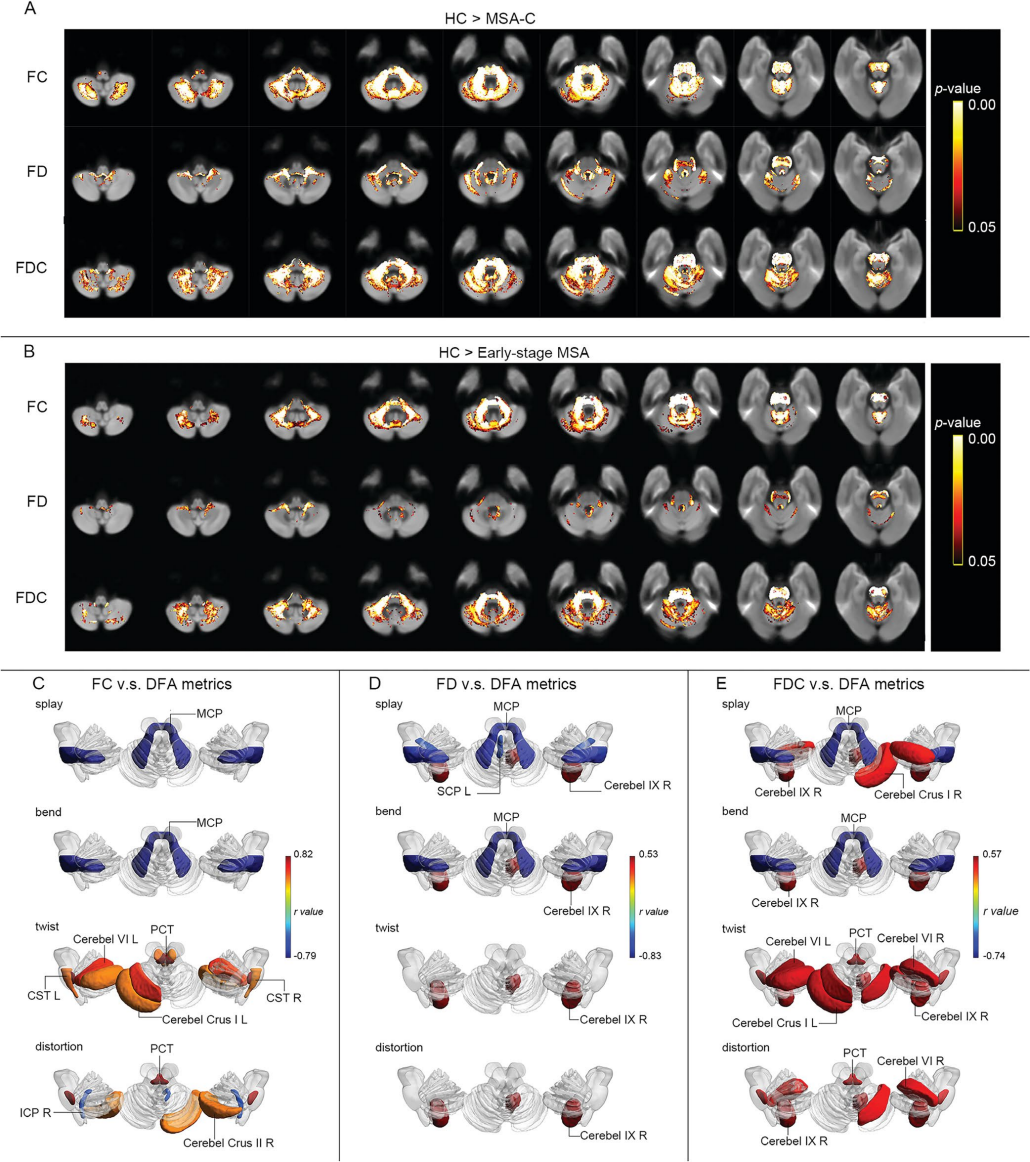
Fixel‐based and geometric white matter pathology in MSA‐C. (A) Metrics of FC, FD, and FDC were derived using fixel‐based analysis described in Supporting Information 1. Streamline segments were cropped from the template tractogram to include only streamline points that correspond to significant fixels (*p* < 0.05, FWE‐corrected). Streamlines were colored by FWE‐corrected *P*‐values for comparison in FC, FD, and FDC. Significant streamlines are displayed across axial slices of the population template map. (B) Fixel‐based analysis showing decreased FC, FD, and FDC in early‐stage MSA‐C compared with HC. Streamline segments were cropped from the template tractogram to include only streamline points that correspond to significant fixels (*p* < 0.05, FWE‐corrected). Streamlines were colored by FWE‐corrected *P*‐values for comparison in FC, FD, and FDC. Significant streamlines are displayed across axial slices of the population template map. (C) The changes in cerebellum and brainstem geometrics in late‐stage MSA‐C patients is significantly correlated with the decrease of FC. (D) The changes in cerebellum and brainstem geometrics in late‐stage MSA‐C patients is significantly correlated with the decrease of FD. (E) The changes in cerebellum and brainstem geometrics in late‐stage MSA‐C patients is significantly correlated with the decrease of FDC. The decline in white matter integrity is reflected by an increase in FA values and a decrease in MD values. The all shown ROIs were colored by the Pearson's *r* values (*p* < 0.05, FDR‐corrected).

FBA revealed widespread significant reductions in FD, FC, and FDC across cerebellar and brainstem regions in MSA‐C patients compared to healthy controls (HC) (Figure 2A). FC declines were most pronounced in bilateral crus II, crus I, I–IV, VIIb, V, VI; vermis VI/VIIIa; right VIIIa; CST; right ICP; MCP; left ML; and PCT. FD reductions selectively affected right crus I, right I–IV, bilateral IX/X, vermis IX, bilateral V/VI, CST (bilateral), MCP, and left SCP. FDC decreases spanned nearly all implicated regions, with strongest effects in crus I/II (bilateral), I–IV/V/VI/IX (bilateral), vermis VI/VIIIa/IX, right VIIb/VIIIa/X, CST (bilateral), MCP, left ML, and PCT. Strikingly, V–VI, crus I–II, and brainstem relay tracts (MCP, CST, PCT) emerged as consistent epicenters of microstructural disruption across all FBA metrics, implicating these hubs in sensorimotor and cerebellar integration deficits characteristic of MSA‐C.

Additionally, FBA revealed widespread white matter degeneration in early‐stage MSA‐C patients compared to healthy controls. Quantitative comparisons demonstrated significant reductions in all three FBA metrics (FC, FD, and FDC) (Figure 2B). Specifically, early‐stage MSA‐C patients exhibited that decreased FC in multiple regions including right cerebellar crus II, bilateral cerebellar crus I, bilateral cerebellar lobules I–IV, vermis VIIIa and VI, right cerebellar lobule VIIb, bilateral cerebellar lobules VI and V, bilateral CST, PCT and MCP. Reduced FD in right cerebellar lobe V and MCP. Compromised FDC in bilateral cerebellar crus II, crus I, lobules I‐IV and VI, bilateral lobule V, vermis VI, bilateral CST, MCP, and PCT.

Notably, no significant differences in FC, FD, or FDC were detected between early‐ and late‐stage MSA‐C patients within cerebellar or brainstem regions, suggesting that fixel‐based microstructural alterations may plateau prior to clinical stage demarcation in cross‐sectional cohorts.

### 3.4 Correlation Analysis

#### 3.4.1 Cross‐Modal Correlations: DFA With FBA Microstructural Metrics

Region‐specific correlations emerged between DFA and FBA metrics in MSA‐C patients (Figure 2C–E). FC correlated inversely with splay and bend in the MCP (*r* = −0.79 to −0.79, *FDR‐corrected p*‐value < 0.001) and distortion in the right ICP (*r* = −0.47, *FDR‐corrected p*‐value = 0.033), but positively with twist in left crus I (*r* = 0.45, *FDR‐corrected p*‐value = 0.038), left VI (*r* = 0.58, *FDR‐corrected p*‐value = 0.004), bilateral CST (*r* = 0.47–0.50, *FDR‐corrected p*‐value < 0.05), and PCT (*r* = 0.82, *FDR‐corrected p*‐value < 0.001) (Figure 2C). FD showed divergent patterns: negative correlations with splay/bend in MCP and left SCP (*r* = −0.57 to −0.83, *FDR‐corrected p*‐value < 0.001), yet positive correlations with splay, bend, twist, and distortion in right IX (*r* = 0.52–0.53, *FDR‐corrected p*‐value < 0.05) (Figure 2D). FDC mirrored FC/FD trends, with MCP splay/bend (*r* = −0.72 to −0.74, *FDR‐corrected p*‐value < 0.001) and right IX metrics (*r* = −0.52 to −0.57, *FDR‐corrected p*‐value < 0.05) inversely linked, whereas crus I, VI, and PCT twist/distortion (*r* = 0.42–0.52, *FDR‐corrected p*‐value < 0.05) exhibited positive associations (Figure 2E).

Critically, elevated splay/bend in brainstem tracts (MCP, ICP) and attenuated twist in cerebellar regions (crus I, VI) correlated with reduced FC/FD/FDC, suggesting that axonal disorganization and loss of microstructural complexity collectively contribute to white matter degeneration in MSA‐C.

#### 3.4.2 Microstructural‐Clinical Linkages: Imaging Biomarkers and Disability Severity

**FIGURE 3:**
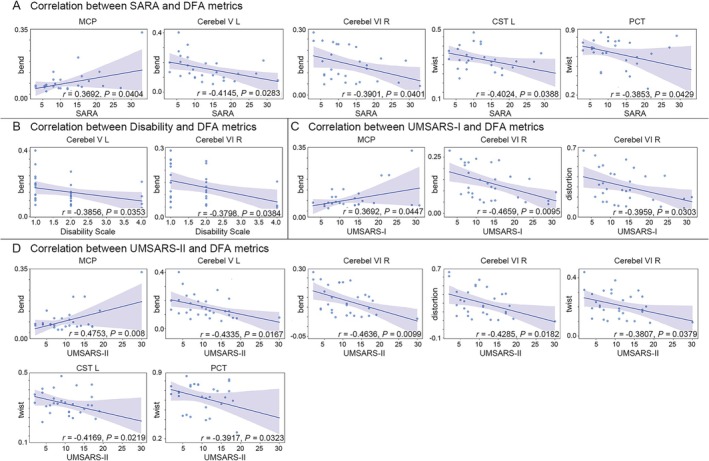
The changes in the geometric characteristics of white matter in MSA‐C patients showed significant correlation with clinical motor abilities. (A) A significant correlation exists between the reduction of white matter geometrics in the cerebellum and brainstem areas of late‐stage MSA‐C patients and the deterioration of SARA. (B) Decrease in bend of white matter in the cerebellum regions of late‐stage MSA‐C patients is related to the increase of disability scale. (C) Changes in geometrics of white matter in the MCP and right cerebellum VI of late‐stage MSA‐C patients is related to the increase of UMSRAS‐I. (D) Changes in geometrics of white matter in the cerebellum and brainstem of late‐stage MSA‐C patients is related to the increase of UMSRAS‐II. White matter DFA metrics were correlated with SARA, UMSARS‐1, and UMSARS‐II using Pearson's correlation, and with the Disability Scale using Spearman's correlation (*p* < 0.05, FDR‐corrected).

Region‐specific geometric alterations in MSA‐C patients correlated with clinical disability severity (Figure 3). SARA scores linked positively with bend in the MCP (*r* = 0.39, *FDR‐corrected p*‐value =0.040) but inversely with bend in left V (*r* = −0.41, *FDR‐corrected p*‐value =0.028), right VI (*r* = −0.39, *FDR‐corrected p*‐value =0.040), and twist in left CST (*r* = −0.40, *FDR‐corrected p*‐value =0.034) and PCT (*r* = −0.39, *FDR‐corrected p*‐value =0.043). Disability scores showed inverse correlations with bend in left V (*r* = −0.39, *FDR‐corrected p*‐value =0.035) and right VI (*r* = −0.38, *FDR‐corrected p*‐value =0.038). UMSARS‐I associated positively with MCP bend (*r* = 0.37, *FDR‐corrected p*‐value =0.045) yet negatively with bend (*r* = −0.47, *FDR‐corrected p*‐value =0.010) and distortion (*r* = −0.40, *FDR‐corrected p*‐value =0.030) in right VI. UMSARS‐II exhibited the strongest associations: positive with MCP bend (*r* = 0.48, *FDR‐corrected p*‐value =0.008) and inverse with bend/twist/distortion in right VI (*r* = −0.46 to −0.38, *FDR‐corrected p*‐value < 0.05), bend in left V (*r* = −0.43, *FDR‐corrected p*‐value =0.017), and twist in left CST (*r* = −0.42, *FDR‐corrected p*‐value =0.022) and PCT (*r* = −0.39, *FDR‐corrected p*‐value =0.032).

Critically, the elevated bend in MCP—consistent with its group‐level increase in MSA‐C—was associated with worse motor and functional disability, while reduced geometric complexity (lower bend, twist, distortion) in cerebellar V–VI and brainstem tracts (CST, PCT) correlated with milder symptoms. This bidirectional pattern implies that brainstem fiber disorganization exacerbates disability, whereas cerebellar geometric simplification may reflect compensatory adaptation or end‐stage deafferentation.

#### 3.4.3 Structural‐Microstructural Coupling in Brainstem Pathology

**FIGURE 4:**
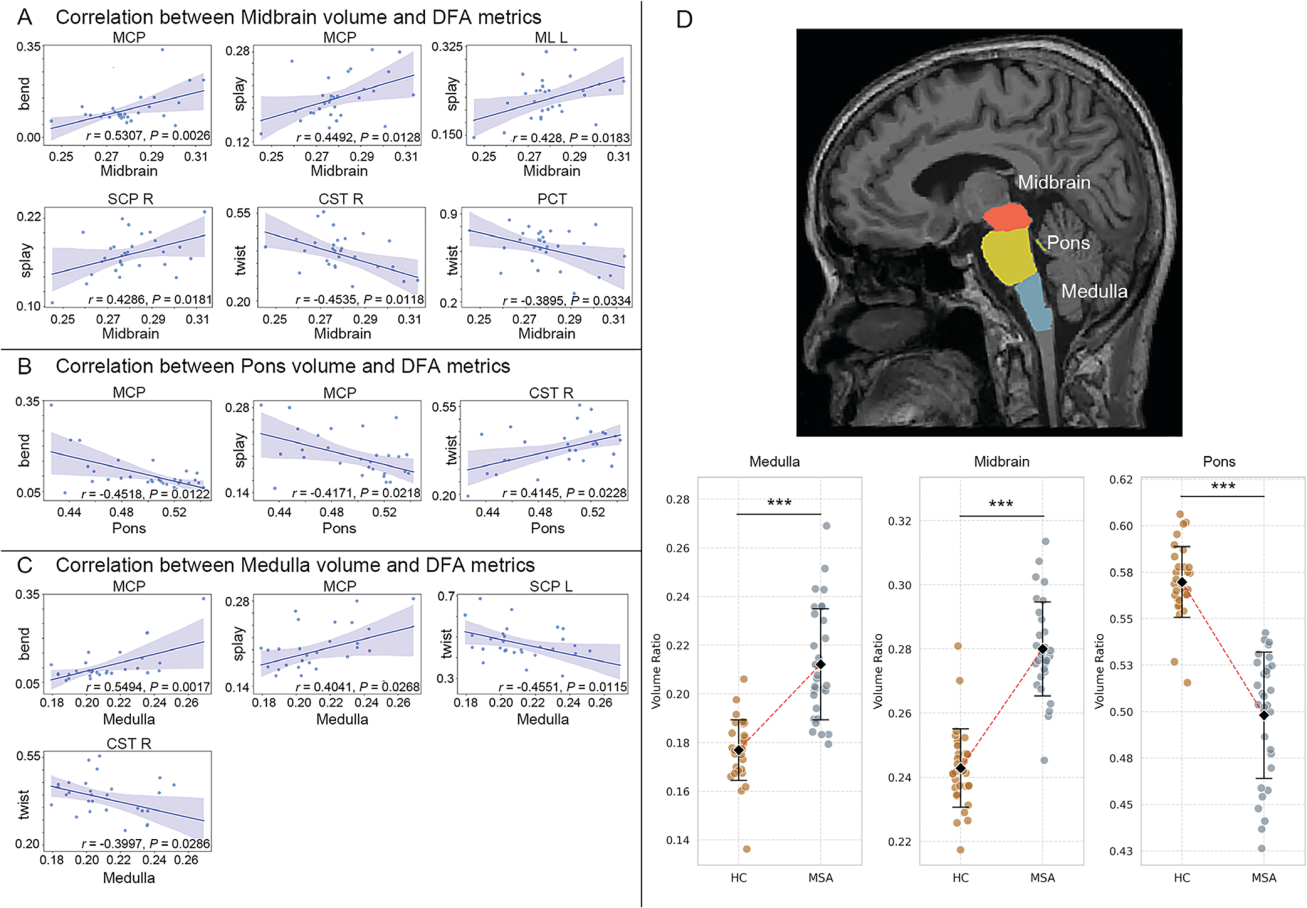
White matter geometry‐brainstem volume relationships in MSA‐C. (A) The increase splay and bend, as well as decline twist, in brainstem white matter in MSA‐C patients is significantly correlated with the expansion of midbrain volume. (B) The increase splay and bend, as well as decline twist, in brainstem white matter in MSA‐C patients is significantly correlated with the atrophy of pons volume. (C) The increase splay and bend, as well as decline twist, in brainstem white matter in MSA‐C patients is significantly correlated with the hypertrophy of medulla volume. White matter DFA metrics were correlated with volume of brainstem subregions using Pearson's correlation (*p* < 0.05, FDR‐corrected). (D) The upper panel shows the anatomical location of medulla, midbrain and pons in brainstem. The down panel shows the volume of medulla and midbrain in MSA‐C patients show significant (*p* < 0.001, FDR‐corrected) larger compared to HC groups, while the volume of pons in MSA‐C group exhibit significant (*p* < 0.001, FDR‐corrected) lower than that in HC group. *** denote FDR‐corrected *p* < 0.001. Orange dots denote HC group and dark gray dots denote MSA‐C group. Means are represented by black solid diamonds, with error bars (black lines) indicating standard deviation.

Geometric abnormalities in MSA‐C patients covaried with brainstem subregional atrophy/expansion (Figure 4). Midbrain (MSA‐C > HC: *t* = −10.97, *FDR‐corrected p*‐value < 0.001) associated positively with bend/splay in the MCP (*r* = 0.45–0.53, *FDR‐corrected p*‐value < 0.05) and splay in left ML (*r* = 0.43, FDR‐corrected *p*‐value = 0.018) and right SCP (*r* = 0.43, *FDR‐corrected p*‐value = 0.018), but inversely with twist in right CST(*r* = −0.45, *FDR‐corrected p*‐value = 0.012) and PCT (*r* = −0.39, *FDR‐corrected p*‐value = 0.033). Pons atrophy (MSA‐C<HC: *t* = 10.38, *FDR‐corrected p*‐value < 0.001) associated negatively with MCP splay/bend (*r* = −0.42 to −0.45, *FDR‐corrected p*‐value < 0.05) yet positively with right CST twist (*r* = 0.41, *FDR‐corrected p*‐value = 0.023). Conversely, medullar expansion (MSA‐C > HC: *t* = −7.65, *FDR‐corrected p*‐value < 0.001) linked positively with MCP bend/splay (*r* = 0.40–0.55, *FDR‐corrected p*‐value < 0.05) but inversely with twist in left SCP (*r* = −0.46, *FDR‐corrected p*‐value = 0.012) and right CST (*r* = −0.40, *FDR‐corrected p*‐value = 0.029).

This bidirectional coupling—elevated splay/bend with medulla/midbrain expansion and reduced twist with pons atrophy—suggests that brainstem geometric derangements (axonal disarray or fiber straightening) parallel region‐specific volume changes. Paradoxically, medullary/midbrain hypertrophy in MSA‐C, contrary to typical neurodegenerative atrophy, may reflect reactive gliosis or edema secondary to α‐synuclein‐mediated neuroinflammation.

Additionally, after normalized to estimated total intracranial volume (eTIV), the three brainstem subregions in MSA‐C patients showed atrophy compared with HC group (medulla: *t* = 3.11; midbrain: *t* = 4.65; pons: *t* = 10.34, all *FDR‐corrected p*‐value < 0.01) (Figure S2A). The lower midbrain volume was significantly negatively correlation with splay, bend and distortion in right ICP (*r* = −0.38 ~ −0.41, *FDR‐corrected p*‐value < 0.05) and twist in left Crus II (*r* = −0.36, *FDR‐corrected p*‐value < 0.05) (Figure S2B).

### 3.5 Machine Learning‐Based Classification of MSA‐C Stages

**FIGURE 5:**
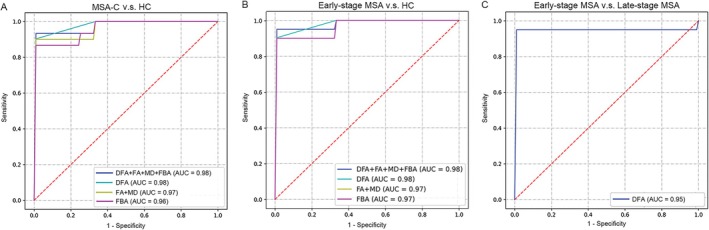
ROC curves. (A) ROC curves for distinguishing MSA‐C from HC using different four feature sets. (B) ROC curves for distinguishing early‐stage MSA‐C from HC using different four feature sets. (C) ROC curve for distinguishing late‐stage MSA‐C from early‐stage MSA‐C patients using twist and distortion in MCP, right CST and PCT as the feature set. In all cases, the area under curve (AUC) is used to assess quality of the ROC curve. Note that the ROC curves for the FA + MD (yellow) and FBA (purple) feature sets are perfectly overlapping, indicating equivalent performance for this specific classification task.

Automated classification using AutoGluon demonstrated high diagnostic accuracy for differentiating HC from MSA‐C and stratifying disease stages (Figure 5A). Models trained on FBA metrics (FC, FD, FDC) achieved an AUC of 0.961 with 93.8% accuracy, while conventional DTI metrics (FA, MD) yielded comparable performance (AUC = 0.967, accuracy = 93.8%). Notably, models based solely on DFA metrics (splay, bend, twist, distortion) outperformed both FBA and DTI (AUC = 0.983, specificity = 96.97%, sensitivity = 93.55%, accuracy = 95.3%), and multimodal integration of DFA + DTI + FBA preserved this high accuracy (AUC = 0.978, accuracy = 95.3%). For distinguishing early‐stage MSA‐C from HC, Models trained on FBA metrics (FC, FD, FDC) achieved an AUC of 0.967 with 93.8% accuracy, while conventional DTI metrics (FA, MD) yielded comparable performance (AUC = 0.967, accuracy = 91.7%). Notably, models based solely on DFA metrics (splay, bend, twist, distortion) outperformed both FBA and DTI (AUC = 0.983, specificity = 96.97%, sensitivity = 93.55%, accuracy = 93.7%), and multimodal integration of DFA + DTI + FBA preserved this high accuracy (AUC = 0.983, accuracy = 93.7%). Notably, in Figure 5B, the ROC curves for the FA + MD (yellow) and FBA (purple) feature sets overlap completely, indicating equivalent performance for this specific classification task (HC vs. early MSA‐C) (Figure 5B). For distinguishing early‐ from late‐stage MSA‐C, DFA‐based features (derived from regions showing stage‐specific geometric alterations) achieved robust classification (AUC = 0.950, sensitivity = 86.67%, specificity = 93.75%, accuracy = 90.32%) (Figure 5C). These results establish white matter geometric signatures as superior biomarkers for MSA‐C diagnosis compared to conventional microstructural metrics, while uniquely enabling disease stage stratification—a critical unmet need in clinical practice (Table 2).

**TABLE 2:** Results of classification using AutoGluon.

Feature	Accuracy	Sensitivity	Specificity	ROC_AUC
HC vs. MSA				
FA + MD + DFA + FBA	0.9531	0.9355	0.9697	0.9778
DFA	0.9531	0.9355	0.9697	0.9833
FA + MD	0.9375	0.9355	0.9394	0.9667
FBA	0.9375	0.9355	0.9394	0.9611
HC vs. early‐stage MSA				
FA + MD + DFA + FBA	0.9375	0.8667	0.9697	0.9833
DFA	0.9375	0.8667	0.9697	0.9833
FA + MD	0.9167	0.8	0.9697	0.9667
FBA	0.9375	0.9333	0.9394	0.9667
Early‐stage MSA vs. Late‐stage MSA				
DFA	0.9032	0.8667	0.9375	0.95

**FIGURE 6:**
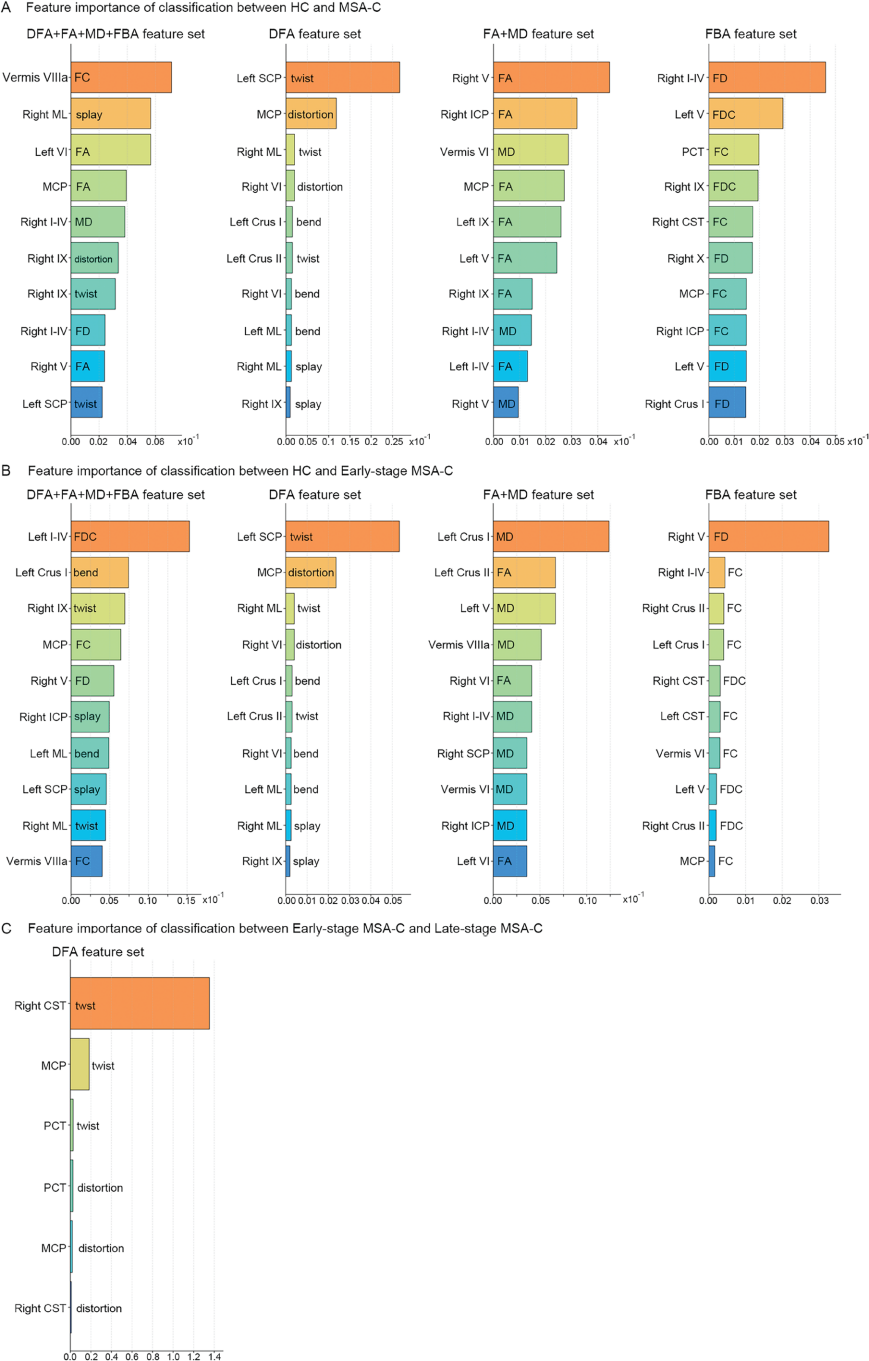
Feature importance analysis for MSA‐C classification performance. (A) Top 10 most discriminative features distinguishing healthy controls (HC) from all MSA‐C patients. (B) Corresponding feature rankings for HC versus early‐stage MSA‐C classification. Notable early markers include left Crus I bend (DFA) and left I–IV FDC (FBA). (C) Progression‐specific features differentiating early vs. late‐stage MSA‐C, dominated by brainstem tract geometric distortions (twist/distortion in MCP, PCT, and CST). All importance scores were normalized to the maximum value within each feature set. In classification of HC versus MSA‐C, the total number of features (dimensionality) for each model was: DFA (*n* = 48), FBA (*n* = 59), FA + MD (*n* = 45), and the multimodal combination (*n* = 152). In classification of HC versus early‐stage MSA‐C, the total number of features (dimensionality) for each model was: DFA (*n* = 42), FBA (*n* = 33), FA + MD (*n* = 35), and the multimodal combination (*n* = 110). In classification of early‐stage MSA‐C versus late‐stage MSA‐C, the total number of features (dimensionality) for each model was: DFA (*n* = 6). CST, corticospinal tract; DFA, director field analysis; FA, fractional anisotropy; FBA, fixel‐based analysis; FC/FDC, fiber cross‐section/fiber density & cross‐section; MCP, middle cerebellar peduncle; MD, mean diffusivity; ML, medial lemniscus; PCT, pontocerebellar tract; SCP, superior cerebellar peduncle.

The feature importance analysis revealed distinct neuroimaging signatures across disease stages (Figure 6). In HC versus MSA‐C classification, combined DFA + FA + MD + FBA metrics identified the splay in right medial lemniscus and FA in right cerebellar lobule V as top discriminators, while FBA metrics highlighted fiber density changes in corticopontocerebellar pathways (Figure 6A). Early‐stage detection (HC vs. early MSA‐C) predominantly involved left cerebellar crus I bend alterations and left lobule I–IV fiber cross‐section reductions (Figure 6B). Disease progression (early vs. late MSA‐C) was best characterized by brainstem‐specific geometric distortions, particularly twist abnormalities in the middle cerebellar peduncle (mean importance score 1.32 ± 0.15) and pontocerebellar tracts (Figure 6C). The consistent emergence of brainstem DFA metrics (twist/distortion) alongside cerebellar FBA/DTI markers suggests complementary pathophysiological processes‐microstructural geometry changes in brainstem circuits coupled with axonal loss in cerebellar projections.

It is important to note that the relatively small sample size in this study may contribute to the observed discretization of performance metrics (e.g., identical accuracy values in Table 2) and the overlapping ROC curves between some feature sets (e.g., FA + MD and FBA in Figure 5B). While the DFA‐based model achieved numerically the highest AUC for the HC vs. MSA‐C classification, its performance was comparable to other modalities. This suggests that the primary advantage of DFA may not solely lie in a dramatic improvement in classification accuracy within this cohort, but rather in its unique ability to provide novel, interpretable geometric biomarkers that capture distinct aspects of white matter pathology complementary to conventional metrics.

## 4 Discussion

This study establishes white matter geometry as a novel biomarker system for MSA‐C through DFA. Three key advances emerge: First, DFA reveals spatially polarized geometric pathology—cerebellar tracts exhibit simplified fiber architecture (reduced splay/bend), while brainstem pathways show dissociated deformation with elevated dispersion yet attenuated twist. Progressive twist reduction in CST, PCT, and MCP tracts distinguishes late from early MSA‐C (AUC = 0.95), performing comparably to or numerically better than conventional diffusion metrics in this cohort. Second, geometric signatures encode distinct degenerative mechanisms: cerebellar simplification correlates with axonal loss (reduced fiber density/cross‐section), whereas brainstem dispersion aligns with midbrain/medulla hypertrophy and ponts atrophy, suggesting region‐specific gliovascular remodeling. Third, multimodal integration of DFA with FBA and FA/MD achieves near‐perfect classification (AUC = 0.98) in this cohort, highlighting its potential as a computational biomarker for disease stratification.

The regionally distinct cerebellar‐brainstem pathology patterns challenge the conventional homogeneity assumption of MSA‐C progression. Our diffusion metrics reveal two fundamental patterns of white matter degeneration: (1) Cerebellar splay/bend reduction likely reflects Wallerian degeneration [24] secondary to Purkinje cell loss [25], consistent with established histopathological evidence of axonal fragmentation and fiber rarefaction [26]. (2) Brainstem dissociation (elevated splay/bend with twist attenuation) corresponds to characteristic histopathological changes including swollen axons and cytoskeletal collapse [27, 28, 29, 30, 31], with brainstem atrophy reflecting neuronal loss in basis pontis nuclei [32, 33, 34, 35].

The spatial distribution of these microstructural changes follows known neuroanatomical vulnerability patterns in MSA‐C, where corticopontocerebellar circuits show progressive twist reduction [36, 37]. While the underlying molecular drivers require further investigation through multimodal approaches (e.g., PET imaging [38]), our geometric signatures provide in vivo markers of system‐specific degeneration. Clinically, cerebellar deformation patterns (crus II/VI) correlate with limb ataxia severity (SARA), while brainstem geometric alterations (MCP/PCT) predict autonomic dysfunction (UMSARS‐I). This dissociation suggests fundamentally distinct pathophysiological processes: cerebellar changes may reflect compensatory capacity exhaustion, whereas brainstem geometry alterations appear more directly linked to symptom generation.

While DFA overcomes conventional DTI's limitations by quantifying three‐dimensional fiber geometry—capturing inter‐voxel orientational changes critical for detecting axonal branching disorganization—this study has inherent constraints. Cross‐sectional design precludes causal links between geometric decay and clinical progression, necessitating longitudinal studies correlating twist attenuation with α‐synuclein PET. Postmortem validation is required to establish direct relationships between specific geometric patterns (e.g., midbrain splay) and histopathological substrates (gliosis/α‐synuclein burden). Nevertheless, the progression‐selective nature of twist decay (AUC = 0.95) and multimodal diagnostic accuracy (AUC = 0.98) position DFA as a valuable tool for investigating MSA‐C pathology. By bridging microstructural collapse, neuroanatomical reshaping, and clinical progression, white matter geometry redefines MSA‐C as a circuit‐specific proteinopathy with immediate implications for trial design and targeted therapies. While Parkinson's disease represents a logical disease control for MSA‐P studies, its inclusion in MSA‐C biomarker validation is less imperative given minimal symptom overlap (cerebellar ataxia vs. parkinsonism) and distinct pathoanatomical substrates (pontocerebellar vs. nigrostriatal degeneration). Future investigations should nevertheless examine whether our geometric metrics could aid MSA‐P/PD differentiation.

## 5 Conclusion

This study establishes white matter geometry as a pivotal biomarker system in MSA‐C, decoding spatially divergent degeneration patterns through director field analysis. Cerebellar fiber simplification (reduced splay/bend) reflects Purkinje cell loss‐driven Wallerian degeneration, while brainstem dissociated geometry (elevated dispersion with reduced twist) tracks α‐synuclein propagation along corticopontocerebellar circuits. Crucially, brainstem twist reduction emerges as a progression‐selective marker, stratifying early and late MSA‐C with high accuracy (AUC = 0.95) and outperforming conventional diffusion metrics. The symptom‐specific correlations—linking motor‐autonomic deficits to brainstem‐cerebellar geometric shifts—redefine MSA‐C as a circuit‐specific proteinopathy. Our multimodal framework, integrating geometry with microstructural and volumetric data (AUC = 0.98), provides a rigorously validated protocol for assessing white matter pathology, with immediate implications for clinical trial design. These geometric signatures demonstrate high translational potential as actionable biomarkers for MSA‐C progression monitoring.

## Author Contributions

H.Z., J.C., and H.L. have contributed to conception and design of the study; H.Z., S.Z., and J.C. have contributed to drafting a significant portion of the manuscript and/or figures; M.Z. participated in reviewing and editing the manuscript; All authors have contributed to acquisition and/or analysis of the data.

## Ethics Statement

This study was approved by the Medical Research Ethics Committee of Xuanwu Hospital of Capital Medical University (LYS[2022]197‐001). All patients and HCs provided written informed consent before participating in the study.

## Conflicts of Interest

The authors declare no conflicts of interest.

## Supporting information


**Figure S1:** DFA metrics and white matter tracts selected. (A) A brief illustration of three types of orientational distortions (i.e., splay, bend, and twist). The figure was revised and approved from Cheng and Basser (2017). (B) Each row shows a synthetic tensor field with DFA index maps (splay, bend, twist, total distortion, FA and MD) calculated from the tensor field. All tensors in the tensor fields share the same shape but have different orientations, resulting in constant FA and MD maps. While the new orientational metrics (splay, bend, twist, and total distortion) could reflect the local spatial orientational distortions. (C) Cerebellum and brainstem regions for voxel‐wise analyses. The cerebellum atlas (upper) and brainstem atlas are from FSL. All voxel‐level statistical comparisons (e.g., DFA metric differences between MSA‐C and HC) were restricted to these anatomically constrained masks to ensure region‐specific hypothesis testing.

**Figure S2:** White Matter Geometry‐Brainstem Volume (normalized to eTIV) Relationships in MSA‐C. (A) The decline splay, bend and distortion in right ICP, as well as decline twist in left Crus II in MSA‐C patients is significantly correlated with the atrophy of midbrain volume. White matter DFA metrics were correlated with volume of brainstem subregions using Pearson's correlation (*p* < 0.05, FDR‐corrected). (D) The volume of medulla, midbrain and pons in MSA‐C patients show significant (*p* < 0.01, FDR‐corrected) lower compared to HC groups. *** denote FDR‐corrected *p* < 0.001, ** denote FDR‐corrected *p* < 0.01. Orange dots denote HC group and dark gray dots denote MSA‐C group. Means are represented by black solid diamonds, with error bars (black lines) indicating standard deviation.

Supporting Information 1–7.

## Data Availability

All data generated and analyzed in the current study were collected from distinct subjects and are available from the corresponding author upon reasonable request.
