# Supplementary figures and images for: Cerebellar and Brainstem White Matter Geometric Alterations in Multiple System Atrophy: A DFA‐Based Biomarker for Disease Staging
Source: CNS Neurosci Ther. 2025 Nov 30;31(12):e70623. doi: 10.1111/cns.70623 (PMC12665615; doi:10.1111/cns.70623)

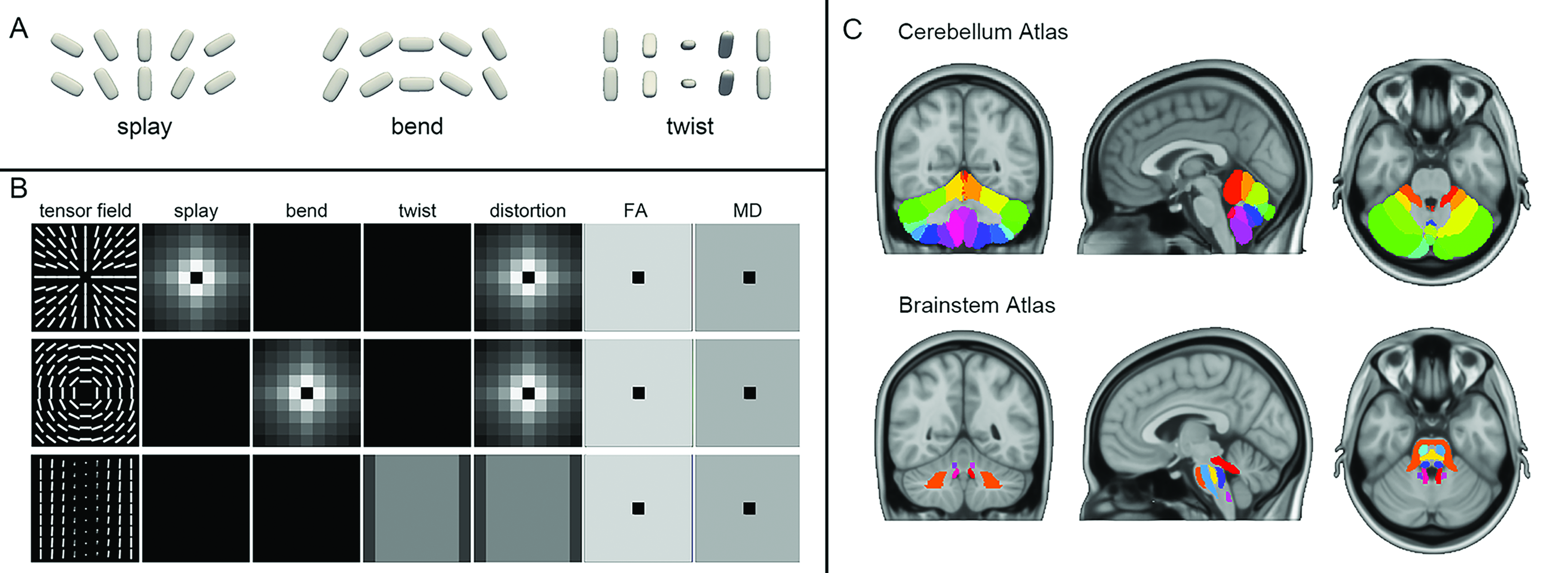

Supplement: Supplementary file 1 — Figure S1: DFA metrics and white matter tracts selected. (A) A brief illustration of three types of orientational distortions (i.e., splay, bend, and twist). The figure was revised and approved from Cheng and Basser (2017). (B) Each row shows a synthetic tensor field with DFA index maps (splay, bend, twist, total distortion, FA and MD) calculated from the tensor field. All tensors in the tensor fields share the same shape but have different orientations, resulting in constant FA and MD maps. While the new orientational metrics (splay, bend, twist, and total distortion) could reflect the local spatial orientational distortions. (C) Cerebellum and brainstem regions for voxel‐wise analyses. The cerebellum atlas (upper) and brainstem atlas are from FSL. All voxel‐level statistical comparisons (e.g., DFA metric differences between MSA‐C and HC) were restricted to these anatomically constrained masks to ensure region‐specific hypothesis testing. [file CNS-31-e70623-s001.tif]

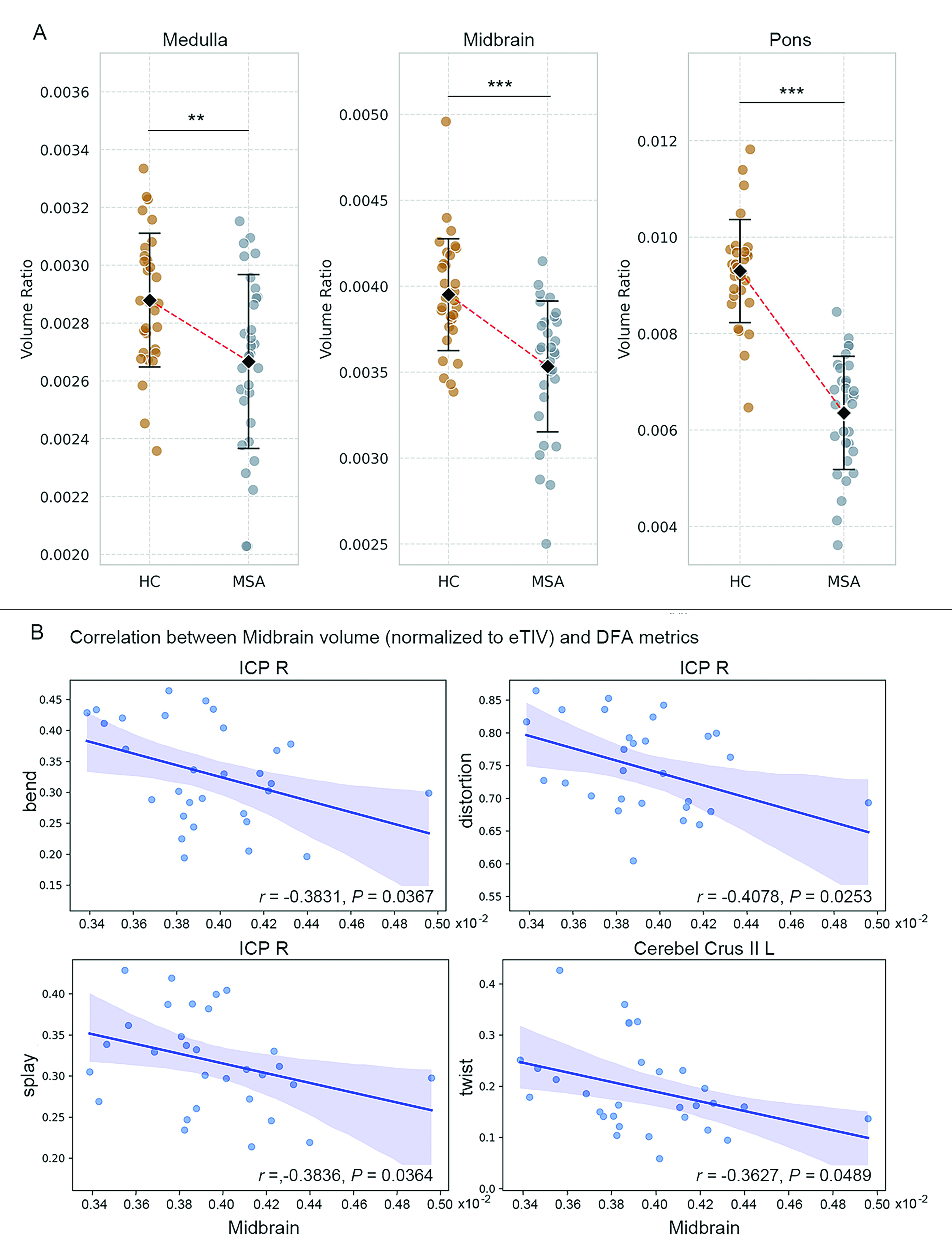

Supplement: Supplementary file 2 — Figure S2: White Matter Geometry‐Brainstem Volume (normalized to eTIV) Relationships in MSA‐C. (A) The decline splay, bend and distortion in right ICP, as well as decline twist in left Crus II in MSA‐C patients is significantly correlated with the atrophy of midbrain volume. White matter DFA metrics were correlated with volume of brainstem subregions using Pearson's correlation (p < 0.05, FDR‐corrected). (D) The volume of medulla, midbrain and pons in MSA‐C patients show significant (p < 0.01, FDR‐corrected) lower compared to HC groups. *** denote FDR‐corrected p < 0.001, ** denote FDR‐corrected p < 0.01. Orange dots denote HC group and dark gray dots denote MSA‐C group. Means are represented by black solid diamonds, with error bars (black lines) indicating standard deviation. [file CNS-31-e70623-s003.tif]
