# Supplementary material for: Cerebellar and Brainstem White Matter Geometric Alterations in Multiple System Atrophy: A DFA‐Based Biomarker for Disease Staging
Source: CNS Neurosci Ther. 2025 Nov 30;31(12):e70623. doi: 10.1111/cns.70623 (PMC12665615; doi:10.1111/cns.70623)
Supplement: Supplementary file 3 — Supporting Information 1–7. [file CNS-31-e70623-s002.docx]

**Supplementary Material 1**

**A brief explanation of the clinical scales:**

**SARA (Scale for the Assessment and Rating of Ataxia)**: A standardized clinical scale (range: 0–40) used to evaluate the severity of cerebellar ataxia. Assesses gait, stance, speech, limb coordination, and oculomotor function, with higher scores indicating worse ataxia.

**Disability Scores:** Refers to functional impairment assessments, which include disease-specific measures (e.g., the Unified Multiple System Atrophy Rating Scale for MSA). In our study, this was based on clinician-rated functional limitations (e.g., mobility, self-care).

**UMSARS-I (Unified MSA Rating Scale, Part I):** A patient/caregiver-reported scale (range: 0–48) assessing non-motor symptoms (e.g., autonomic dysfunction, speech, swallowing) and daily activities in multiple system atrophy (MSA).

**UMSARS-II (Unified MSA Rating Scale, Part II):** A clinician-rated motor examination scale (range: 0–56) evaluating motor impairment in MSA, including parkinsonism, cerebellar ataxia, and gait/balance deficits.

**Supplementary Material 2**

**SARA Score Sheet**

| **Item** | **Examination** | **Score Range** | **Scoring Criteria** |
| --- | --- | --- | --- |
| **1. Gait** | 8-meter walk (observed) | 0–8 | **0**: Normal;  **2**: Mild imbalance (uses wall support);  **4**: Marked imbalance (requires intermittent support);  **6**: Severe imbalance (requires constant support);  **8**: Unable to walk |
| **2. Stance** | Feet together, eyes open → feet together, eyes closed → tandem stance | 0–6 | **0**: Normal (≥30s);  **1**: Sway with eyes open;  **2**: Falls with eyes closed;  **3**: Unstable in tandem;  **4**: Unable tandem;  **6**: Unable feet together |
| **3. Sitting** | Sitting without back support (arms folded) | 0–4 | **0**: Normal (≥10s);  **1**: Slight trunk sway;  **2**: Moderate sway;  **4**: Unable without arm support |
| **4. Speech** | Spontaneous speech or reading | 0–6 | **0**: Normal;  **2**: Mild dysarthria (slurring);  **4**: Moderate dysarthria;  **6**: Severe dysarthria/aneffective speech |
| **5. Finger Chase** | Finger-to-nose test (eyes open) | 0–4 | **0**: No dysmetria;  **1**: Mild dysmetria (overshoot <5cm);  **2**: Moderate dysmetria (overshoot 5–10cm);  **3**: Severe dysmetria (overshoot >10cm);  **4**: Unable perform |
| **6. Nose-Finger Test** | Finger-to-nose test (eyes closed) | 0–4 | *Same as Item 5* |
| **7. Fast Hand Movements** | Pronation/supination of hands (10s each side) | 0–4 | **0**: Normal rhythm/amplitude;  **1**: Mild irregularity;  **2**: Moderate irregularity;  **3**: Severe irregularity;  **4**: Unable perform |
| **8. Heel-Shin Slide** | Heel down shin (supine position, each leg) | 0–4 | **0**: Smooth;  **1**: Mild deviation;  **2**: Moderate deviation (off shin <50%);  **3**: Severe deviation (off shin >50%);  **4**: Unable perform |
| **Total Score** |  | **0–40** | *Higher scores indicate more severe ataxia* |

**Supplementary Material 3**

**Supplementary Table 1 The information about cerebellum and brainstem tracts.**

| **Cerebellum** | | | | **Brainstem** | |
| --- | --- | --- | --- | --- | --- |
| **Index** | **Name** | **Index** | **Name** | **Index** | **Name** |
| **1** | **Left I-IV** | **15** | **Vermis VIIb** | **1** | **MCP** |
| **2** | **Right I-IV** | **16** | **Right VIIb** | **2** | **PCT** |
| **3** | **Left V** | **17** | **Left VIIIa** | **7** | **Right CST** |
| **4** | **Right V** | **18** | **Vermis VIIIa** | **8** | **Left CST** |
| **5** | **Left VI** | **19** | **Right VIIIa** | **9** | **Right ML** |
| **6** | **Vermis VI** | **20** | **Left VIIIb** | **10** | **Left ML** |
| **7** | **Right VI** | **21** | **Vermis VIIIb** | **11** | **Right ICP** |
| **8** | **Left Crus I** | **22** | **Right VIIIb** | **12** | **Left ICP** |
| **9** | **Vermis Crus I** | **23** | **Left IX** | **13** | **Right SCP** |
| **10** | **Right Crus I** | **24** | **Vermis IX** | **14** | **Left SCP** |
| **11** | **Left Crus II** | **25** | **Right IX** |  |  |
| **12** | **Vermis Crus II** | **26** | **Left X** |  |  |
| **13** | **Right Crus II** | **27** | **Vermis X** |  |  |
| **14** | **Left VIIb** | **28** | **Right X** |  |  |

**Supplementary Material 4**

**Fixel-based analysis**

The fixel-based analysis utilized in this study comprises a detailed sequence of image preprocessing and processing steps. Preprocessing of diffusion-weighted images included denoising and unringing, correction for motion and distortion, bias field correction. Intensity normalization across subjects was performed by deriving scale factors from the median intensity in select voxels of white matter, grey matter, and CSF in b0 images. The methodology then includes the computation of an average white matter response function, followed by the upsampling of diffusion images to a voxel size of 1 mm^3^. Upsampled brain mask images were also computed at this stage.

Following these preprocessing steps, fibre orientation distributions (fODFs) were computed using Single-Shell, 3-Tissue Constrained Spherical Deconvolution (SS3T-CSD), with group averaged response functions for white matter, grey matter, and CSF. Spatial correspondence was achieved by first generating a group-specific population template with an iterative registration and averaging approach using fODF images from all participants. Each participant’s fODF image was then registered to the template via a FOD-guided non-linear registration. A tractogram was generated using whole-brain probabilistic tractography on the population template. Twenty million streamlines were first generated, and these were subsequently filtered to 2 million streamlines using the SIFT (spherical-deconvolution informed filtering of tractograms) algorithm to reduce reconstruction biases.

The fixel-based metrics of fibre density (FD), fibre-bundle cross-section (FC) and a combined measure of fibre density and cross-section (FDC) were derived for each participant across all white matter fixels. To identify regions with altered FD, FC and FDC across all three groups, statistical comparisons between two groups were performed at each white matter fixel by a General Linear Model, comparing (i) MSA patients versus healthy controls; (ii) late-stage MSA patients versus healthy controls; a. Connectivity-based smoothing and statistical inference was performed using connectivity-based fixel enhancement (CFE), using 2 million streamlines from the template tractogram, with default smoothing parameters (smoothing = 10 mm full-width at half-maximum, C = 0.5, E = 2, H = 3). Family-wise error (FWE)-corrected *p*-values were then assigned to each fixel using non-parametric permutation testing over 5000 permutations.

**Supplementary Material 5**

**Fixel-based metrics**

The fixel-based metrics of fibre density (FD), fibre-bundle cross-section (FC) and a combined measure of fibre density and cross-section (FDC) for each participant across all white matter fixels provide distinct physiological insights: (A) FD reflects intra-axonal fiber density within a voxel, serving as a marker of axonal integrity and myelination; (B) FC captures macroscopic changes in fiber bundle cross-sectional area, often linked to atrophy or growth; (C) FDC (the product of FD and FC) integrates micro- and macrostructural information, offering a composite measure of white matter "health" sensitive to neurodegenerative or developmental changes. Together, these metrics enable nuanced investigations of white matter pathology, plasticity, and connectivity in neurological disorders. FD measures the local density of fibers within a fixel, indicating the quantity and compactness of nerve fibers in a specific direction. FC estimates the cross-sectional area of fiber bundles, reflecting changes in their morphology due to disease or over time. FDC combines both FD and FC to provide a comprehensive overview of overall fiber health, including both quantity and structural size.

**Supplementary Material 6**

**Freesurfer Process**

Freesurfer processing for volumetric T1-weighted images included: motion correction, brain extraction and removal of non-brain tissue using a hybrid watershed/surface deformation procedure; automated spatial transformation and WM segmentation of subcortical volumetric structures; intensity normalization, tessellation of GM/WM boundary and automated topology correction; and surface deformation following intensity gradients to optimally place GM/WM and GM/CSF borders at the location where the greatest shift in intensity defines the transition to the other tissue class. Image outputs from each stage of Freesurfer processing were visually inspected and edited by an experienced imaging analyst. Quantitative estimates were derived in a large set of spatially distinct brain tissue that covered the whole brainstem. The three subregions of the brainstem include the medulla, midbrain, and pons. The three subregions of brainstem volumes were corrected/normalized by dividing brainstem volume for each participant (e.g., medulla volume = medulla volume / brainstem volume). Additionally, the three subregions of the brainstem were also normalized to estimated total intracranial volume (eTIV).

**Supplementary Material 7**

**AutoGluon Prediction Workflow**

AutoGluon-Tabular processes structured data through an integrated multi-stage pipeline: Upon receiving input features (e.g., FA/MD, DFA, and FBA metrics), it first applies automated feature engineering: numerical features are Z-score normalized, categorical variables (e.g., brain region labels) undergo entity embedding encoding [1], and text fields convert to n-gram vectors. Crucially, all features enter the system with equal initial weighting—no predefined feature weights are imposed. For model construction, AutoGluon initializes six heterogeneous base learners: LightGBM [2], CatBoost [3], neural networks (with categorical embeddings and skip-connections), Random Forests, Extremely Randomized Trees, and K-Nearest Neighbors—all using fixed hyperparameters (no tuning). Each base model undergoes 10-fold stratified bagging: the training set is split into 10 folds preserving class ratios, with 9 folds training the model and the excluded fold generating out-of-fold (OOF) predictions; this repeats for all folds to create complete OOF prediction matrices. These OOF predictions then feed into the multi-layer stacking ensemble: the stacking layer receives concatenated inputs of original features + all base models' OOF predictions, and trains stacker models (same architectures as base learners) similarly via bagged OOF. Finally, the Ensemble Selection algorithm [4] assigns performance-based weights (summing to 1) to stacker outputs—optimized by validation AUC (eval_metric='roc_auc')—where stronger models receive higher weights (e.g., w₁=0.6 for LightGBM vs. w₂=0.4 for neural nets), producing probabilistic predictions. For test set inference, base models' bagged copies predict on test data, their averaged outputs concatenate with test features for stacker input, and the weighted ensemble generates final labels. Thus, while features enter equally, their ultimate influence emerges via model-specific learning and AUC-optimized ensemble weighting—not manual feature weighting.

**Reference**

1. Guo C, Berkhahn F. Entity embeddings of categorical variable. arXiv, 2016. H2O.ai. H2O AutoML, 2017. URL <http://docs.h2o.ai/h2o/latest-stable/h2o-docs/automl.html>. H2O version 3.30.0.1.
2. Ke G, Meng Q, Finley T, Wang T, et al. LightGBM: A highly efficient gradient boosting decision tree. In NIPS, 2017.
3. Prokhorenkova L, Gusev G, Vorobev A, et al. CatBoost: unbiased boosting with categorical features. In NeurIPS, 2018.
4. Caruana R, Niculescu-Mizil A, Crew G, & Ksikes A. Ensemble selection from libraries of models. In ICML, 2004
